# Nontuberculous mycobacteria in Denmark, incidence and clinical importance during the last quarter-century

**DOI:** 10.1038/s41598-017-06931-4

**Published:** 2017-07-27

**Authors:** Thomas S. Hermansen, Pernille Ravn, Erik Svensson, Troels Lillebaek

**Affiliations:** 1 International Reference Laboratory of Mycobacteriology, Statens Serum Institut, Copenhagen, Denmark; 2 Department of Thoracic Medicine and Infectious Disease, Nordsjaelland Hospital, Copenhagen University Hospital, Hillerød, Denmark

## Abstract

Disease caused by nontuberculous mycobacteria (NTM) is reported to increase due to an ageing population and a rise in the proportion of immunosuppressed patients. We did a retrospective cohort study of NTM-disease in the Danish population through a quarter-century to determine the disease burden and trends in annual incidence rates. 524,119 clinical specimens were cultured for mycobacteria from 1991 through 2015 at the International Reference Laboratory of Mycobacteriology in Denmark. Among these, 8,227 NTM strains were identified from 3,462 patients and distributed according to microbiological disease criteria. We observed no increase in NTM disease incidence or proportion of patients with positive NTM cultures during the study period (Quasi-Poisson regression, p = 0.275 and 0.352 respectively). Annual incidence rates were 1.20/10^5^ for definite NTM disease, 0.49/10^5^ for possible NTM disease and 0.88/10^5^ for NTM colonization. The incidence rate of NTM disease was highest in children aged 0-4 years (5.36/10^5^/year), predominantly with cervical *Mycobacterium avium* complex (MAC) adenitis. Surprisingly, based on more than half a million clinical specimens cultured for mycobacteria in Denmark through 25 years, the NTM disease burden and trend in incidence in the Danish population has not increased opposed to numerous internationals reports.

## Introduction

Non-tuberculous mycobacteria (NTM) are ubiquitous in the environment worldwide^1, 2^. It’s a heterogeneous group of opportunistic pathogens causing human disease predominantly in immuno-compromised individuals and patients with underlying disease such as bronchiectasis and cystic fibrosis^3^. Clinical disease caused by NTM can be very diverse and present with pulmonary lesions and involve extra-pulmonary sites such as lymph nodes, skin and soft tissue, bones and joints. NTM disease can also mimic infection caused by *Mycobacterium tuberculosis*. Both pulmonary NTM colonization and NTM disease have a five-year mortality of approximately 40%, reflecting the high level of comorbidity and/or immune suppression in these patients^4^.

Several studies have reported an increase in both NTM disease and NTM colonization in humans during the last decades^5–10^. The increase in NTM disease has been attributed to different factors such as improved culturing techniques and greater disease awareness, but it has also been attributed to a true increase in disease incidence as a result of increased life expectancy and an increasing proportion of the population experiencing immunosuppression due to medication or suffering from immune-modulating comorbidities such as e.g. diabetes mellitus and chronic obstructive pulmonary disease (COPD)^11–13^.

The majority of studies assessing the significance of NTM colonization and disease have some important limitations. First, the true clinical impact of NTM infections is difficult to assess due to challenges in discriminating between disease and colonization^13^. Second, in most countries, NTM diseases are not notifiable, thus population data are not routinely collected, and incidence rates are based mainly on the numbers of NTM isolated. Finally, the majority of studies have been conducted in localized geographic regions within countries, sometimes based on few observations and/or short observation period, limiting the overall generalizability of results^5, 8, 10, 14, 15^.

In Denmark, for almost one century, the culturing of mycobacteria has been centralized to one laboratory only, the International Reference Laboratory of Mycobacteriology (IRLM) in Copenhagen. This, allows a very comprehensive and exhaustive evaluation of NTM disease burden and trends in annual incidence rates during decades based on microbiological criteria.

## Material and Methods

### Data sources and study design

Retrospective nationwide cohort study including data from IRLM, Denmark, on all patients with a positive NTM culture from January 1991 through December 2015. IRLM is the only laboratory in Denmark which routinely cultivates mycobacteria. The first positive NTM culture per patient was used for incidence rate calculations, whereas the total number of cultures per patient was used for allocating patients into disease categories. If a patient was culture-positive for more than one NTM species during the study period, only the first NTM was included in the study (n = 41, 1.2% of the patients).

### Description of variables

Patients were classified into three NTM disease categories using the modified American Thoracic Society/Infectious Diseases Society of America (ATS/IDSA) 2007 criteria based on microbiological data only^13^: definite NTM disease, possible NTM disease, and NTM colonization. This method has been validated by Andrejak *et al*. in 2010^4^. In addition to the modified ATS/IDSA criteria focusing on pulmonary cases, we included patients with NTM cultured from extra-pulmonary sites as definite NTM disease cases in line with Freeman *et al*.^16^.

Patients were considered to have “definite NTM disease” if they had; A) more than three positive specimens, B) three positive specimens including one or more positive specimen obtained by bronchoscopy, C) three positive specimens including one or more positive bronchial wash, bronchial biopsy or pleural effusion, D) at least one positive specimen from a lung biopsy, or E) a positive specimen from an extra-pulmonary location. Patients were considered to have “NTM colonization”, if they had only one positive NTM specimen, unless this specimen was from; a bronchial wash, a lung or bronchial biopsy, or an extra-pulmonary location. The remaining patients were categorized as “possible NTM disease” based on their fulfilment of microbiological ATS/IDSA criteria of NTM disease without any available clinical data, expect for patients with only one positive culture for *M. gordonae* from a bronchial wash, whom were classified as NTM colonization.

Gastric lavage aspirations were excluded from our study because they were considered a result of contamination. The majority of cultures (78%) contained *M. gordonae* and were from 1998 and 1999, where tap water was used for gastric aspirations resulting in a surge in cultures without clinical relevance.

The patients were divided into three groups; a) Patients with respiratory samples were classified as pulmonary NTM, b) patients with samples from non-respiratory sites (including pleura) were classified as non-pulmonary NTM, and c) patients with samples from both pulmonary and non-pulmonary sites were classified as multifocal NTM.

Patient age was calculated from the unique Personal Identification Number in the Danish Civil Registration System^17^. The incidence rates were stratified in five-year age intervals standardized to the population demographics^18^. This standardization ensured that disease events were not disproportionately weighted in extreme age groups. Annual incidence rates were calculated based on an increase in the Danish population from 5,148,000 in 1991 to 5,658,000 in 2015.

NTM species identification was carried out by either AccuProbe (Gen-Probe, San Diego, CA) and supplementary biochemical tests (1991–2001), InnoLipa species (InnoGenetics, Ghent, Belgium, 2001–2012) or GenoType Mycobacterium CM/AS (Hain Lifescience, 2012–2015). Throughout the study period, 16 S DNA sequencing using primers directed at the hypervariable region A was used as a supplement when species were not identifiable by other methods^19^. The most common NTM species encountered were included individually in the analysis, the remaining species were referred to as “Others”.

### Statistics

The quantitative variables were described as mean and standard deviation of normally distributed variables and as median and interquartile range of non-normally distributed variables. The qualitative variables were described as numbers and percentages out of the total.

Incidence rates were found by summarising the mid-year population estimates for the Danish population each year during the study period. To evaluate a trend in annual incidence rates of NTM disease during the study period, we constructed a Quasi-Poisson regression model. This was chosen to allow for over-dispersion of data. We corrected for serial correlation by adding the observed incidence rate the previous year as explanatory variable in the model. Relative risks (RR) with 95% confidence intervals (CI) for definite or possible NTM disease in the different age groups were calculated. Multiple group comparisons were performed using the non-parametrically Kruskal-Wallis rank sum test to investigate the association between patient age and year of NTM culture according to gender. The χ^2^ test for trend in proportions was used to evaluate the association between year of culture and the proportions of NTM colonization versus NTM definite and possible disease. A non-significant trend test indicates no linear trend towards an increase or decrease during the study period. A significance level of p < 0.05 was considered significant. For the statistical analysis, R version 3.1.1 was used.

### Ethics

The study was approved by the Danish Data Protection Agency J.no.: 2012-54-0100. Permission from an ethical committee was not required.

## Results

From 1991 through 2015, IRLM performed 524,119 cultures. Among these, 8227 (1.6%) were NTM culture positive, retrieved from 3,462 individuals. Using microbiological criteria; 1,618 (47%) patients had definite NTM disease, 654 (19%) had possible NTM disease and 1,190 (34%) NTM colonization. 2,391 (69.1%) of samples were from pulmonary sites, 926 (26.7%) from non-pulmonary sites, and 145 (4.2%) from multifocal sites. In total, 51 different species were isolated. For frequency of NTM species according to disease category, age, localization of disease, and sex, see Table 1. Annual numbers and proportions of patients in the three disease categories are shown in Table 2. The annual number of positive cultures in the population did not increase or decrease during the study period (p = 0.836, data not shown).

**Table 1:** NTM frequency according to disease category, age, sample localization, and sex.

NTM species	Total	Definite NTM disease	Possible NTM disease	NTM coloni-zation	Age Median [IQR]	Localisation Pulmonary %	Localisation Non pulmonary %	Sex male%
	n	n	n	n				
*M. abscessus/chelonae*	195	93	37	65	46.6 [45.2]	82.6	15.9	54.4
*M. avium complex*	1,757	1,093	403	261	55.0 [59.1]	56.9	35.9	52.6
Adults aged 15+*	1,309	652	400	257	64.2 [26.0]	75.1	15.5	56.3
Children aged 0–14^†^	448	441	3	4	2.5 [2.0]	3.8	95.3	41.7
*M. gordonae*	527	0	0	527	65.9 [21.9]	94.1	5.5‡	58.4
*M. kansasii*	55	34	15	6	50.0 [36.4]	83.6	12.7	58.2
*M. malmoense*	139	97	23	19	59.5 [42.0]	71.2	28.8	55.4
*M. marinum*	100	100	0	0	44.4 [27.4]	1.0	98.0	72.0
*M. fortuitum/peregrinum*	123	15	24	84	59.4 [31.0]	91.9	8.1	59.3
*M. xenopi*	140	61	42	37	63.6 [21.6]	95.0	2.9	60.0
Others^§^	426	125	110	191	62.1 [25.5]	81.0	17.5	55.8
Total	3,462	1,618	654	1,190	58.5 [37.2]	69.1**	26.7**	55.4

^*^Among adults with MAC, 9.4% (123) were from a multifocal origin (total 1,309). ^†^Among children 0.9% (4) were from a multifocal origin (a total of 448/561 samples were MAC). ^‡^Of these, 76% (22/29) of specimens were from urine or fecal samples. ^§^Others including uncommon mycobacteria and mycobacteria not identified (a complete list is available in Table 4). **4.2% of samples were from a multifocal origin.

**Table 2:** Annual number of NTM isolates in the study period.

	1991	1992	1993	1994	1995	1996	1997	1998	1999	2000	2001	2002	2003
Definite disease (%)	75 (66)	60 (69)	111 (72)	80 (56)	74 (56)	81 (56)	43 (44)	36 (22)	57 (26)	49 (40)	48 (40)	47 (56)	51 (51)
Possible disease (%)	24 (21)	21 (24)	23 (15)	22 (15)	24 (19)	24 (17)	13 (13)	18 (11)	18 (8)	10 (8)	16 (13)	16 (19)	18 (18)
Colonization (%)	15 (13)	6 (7)	21 (14)	42 (29)	30 (23)	40 (28)	41 (42)	109 (67)	143 (66)	62 (51)	55 (46)	21 (25)	30 (30)
Total	114	87	155	144	128	145	97	163	218	121	119	84	99
	**2004**	**2005**	**2006**	**2007**	**2008**	**2009**	**2010**	**2011**	**2012**	**2013**	**2014**	**2015**	
Definite disease (%)	50 (45)	93 (64)	49 (47)	57 (46)	63 (45)	77 (44)	72 (42)	78 (55)	85 (52)	65 (38)	53 (36)	64 (34)	
Possible disease (%)	20 (18)	20 (14)	20 (19)	23 (18)	30 (21)	23 (13)	40 (23)	37 (26)	31 (19)	35 (21)	54 (37)	75 (39)	
Colonization (%)	41 (37)	32 (22)	36 (34)	45 (36)	48 (34)	75 (43)	61 (35)	27 (19)	48 (29)	70 (41)	39 (27)	52 (27)	
Total	111	145	105	125	141	175	173	142	164	170	146	191	

Percentages row wise each year (percentages differ 99–101 due to rounding).

### NTM incidence rates

**Figure 1:**
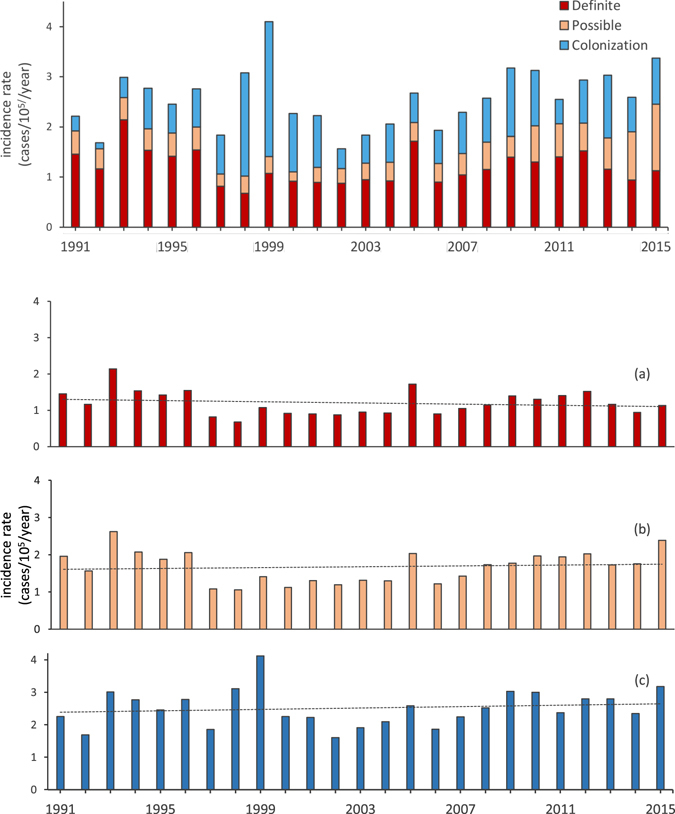
Annual incidence rates of definite NTM disease, possible NTM disease and NTM colonization. (**a**) Annual incidence rates of definite NTM disease, (**b**) annual incidence rates of definite and possible NTM disease and (**c**) annual incidence rates of total number of patients with a positive NTM culture. Quasi-Poisson generalized linear model found no trend towards an increase or decrease in the study period for definite NTM disease incidence (**a**, p = 0.674), definite and possible NTM disease incidence (**b**, p = 0.275) and patients with a positive NTM culture (**c**, p = 0.352).

During the study period, we found no significant trend when calculating the overall incidence rate for patients with a positive NTM culture (definite NTM disease, possible NTM disease and NTM colonization combined, p = 0.3516, Fig. 1). The overall annual incidence rate was 2.57/10^5^; 1.20/10^5^ for definite NTM disease, 0.49/10^5^ for possible NTM disease, and 0.88/10^5^ for NTM colonization. Also, we found no significant trend for definite NTM disease (p = 0.674) (Fig. 1) or for the combined incidence rates of definite NTM disease and possible NTM disease (p = 0.275) (Fig. 1). Similarly, the incidence rate for patients with NTM colonization did not increase or decrease during the study period (p = 0.663).

### NTM and age

**Figure 2:**
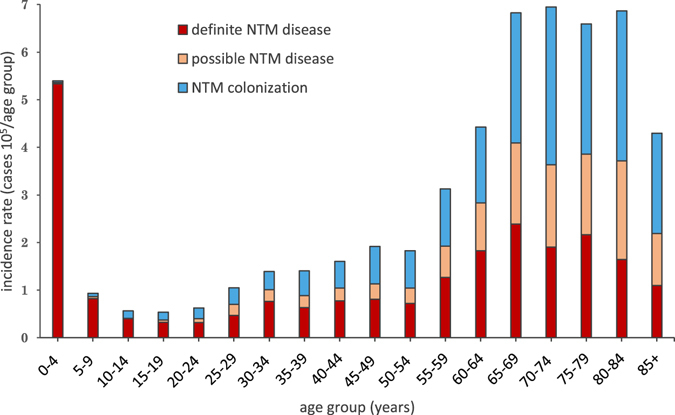
Incidence rates of NTM disease categories in the study period standardized to age groups.

Age-standardized incidence rates revealed significant differences according to age groups (Table 3 and Fig. 2). Among the adult population aged 15 years and above, the incidence of definite NTM disease, possible NTM disease, and NTM colonization were 0.99/10^5^/year, 0.59/10^5^/year and 1.06/10^5^/year, respectively. Thus, a large proportion of NTM cultures represented possible disease (647/2,901; 22%) and colonization (1,167/2,901; 40%). Reversely, among children, the majority of positive NTM cultures represented definite disease (530/561; 95%) (Fig. 2). The 0–4 years group had an incidence rate of NTM disease of 5.36/10^5^/year and the relative risk (RR) of a positive NTM culture among children aged 0–4 years was 10.0 (95% CI [7.3-13.8]) compared to the reference age group with the lowest annual incidence; adolescents aged 15–19 years. Among adults, age-adjusted incidence rates were at a steady level among the age groups 15–54 years (0.54–1.92/10^5^), increasing from age 55 years. The median age of all adults (15 + years) increased during the study period from 49.4 (IQR 34.1) in 1991 to 68.2 (IQR 19.2) in 2015 (p < 0.00001, Kruskal-Wallis) (data not shown in table).

**Table 3:** Age standardized, annual incidence rates per 10^5^ population and origin of NTM culture.

Age, years	All NTM cultures		Definite NTM disease		Possible NTM disease		NTM Colonization		Localization*		
	n	Annual incidence /10^5^	n	Annual incidence/10^5^	n	Annual incidence/10^5^	n	Annual incidence/10^5^	%Pulmonary %Multifocal %Non-pulmonary		
0–4	429	5,40	424	5,34	2	0,03	3	0,04	2	1	97
5–9	83	0,93	73	0,82	4	0,04	6	0,07	13	1	85
10–14	47	0,56	33	0,40	1	0,01	13	0,16	71	2	27
15–19	42	0,54	25	0,32	4	0,05	13	0,17	72	2	26
20–24	51	0,62	26	0,32	7	0,09	18	0,22	63	8	28
25–29	96	1,05	43	0,47	21	0,23	32	0,35	46	19	35
30–34	137	1,39	75	0,76	24	0,24	38	0,39	45	17	38
35–39	138	1,40	62	0,63	25	0,25	51	0,52	51	15	34
40–44	151	1,60	73	0,77	25	0,27	53	0,56	58	11	31
45–49	178	1,92	75	0,81	30	0,32	73	0,79	64	11	25
50–54	182	1,83	72	0,72	32	0,32	78	0,78	77	6	17
55–59	257	3,13	104	1,27	54	0,66	99	1,20	87	2	11
60–64	286	4,42	118	1,83	65	1,01	103	1,59	89	1	10
65–69	377	6,83	132	2,39	94	1,70	151	2,73	92	2	5
70–74	365	6,95	100	1,90	91	1,73	174	3,31	94	1	5
75–79	311	6,60	102	2,16	80	1,70	129	2,74	94	1	5
80–84	222	6,87	53	1,64	67	2,07	102	3,16	94	1	6
85+	110	4,30	28	1,09	28	1,09	54	2,11	92	0	8
Total	3,462	2.57	1,618	1.20	654	0.49	1,190	0.88	69	4	27

NA: Not applicable. *Of all NTM cultures.

### NTM species

The five most commonly identified species in the Danish population were MAC (n = 1,757), *M. gordonae* (n = 527), *M. abscessus/chelonae* (n = 195), *M. malmoense* (N = 139), and *M. xenopi* (n = 140) (Table 1). There was no difference in male to female ratio compared to the overall distribution, except for *M. marinum* (72% males). MAC accounted for 51% (1,757/3,462) of all NTM isolates and 68% (1,093/1,618) of all definite NTM disease cases, as the most commonly identified species. MAC was responsible for 80% (448/561) of all isolates in children aged 0–14 years, and 98% of these children had definite disease (Table 1). The annual incidence rate of definite NTM disease with MAC among children aged 0–4 was 4.6/10^5^. A complete list of all species in the study period is available in Table 4.

**Table 4:** NTM species cultured from human specimens at IRLM 1991–2015, n = 51. (Uidentified mycobacteria n = 167).

*M. abscessus* (*131*)	*M. interjectum* (*17*)
*M. agri* (*4*)	*M. intracellulare* (*233*)
*M. arosiense* (*3*)	*M. kansasii* (*55*)
*M. arupense* (*1*)	*M. lentiflavum* (*8*)
*M. avium* (*1520*)	*M. malmoense* (*139*)
*M. bohemicum* (*6*)	*M. marinum* (*100*)
*M. branderi* (*5*)	*M. mucogenicum* (*8*)
*M. brisbanense* (*1*)	*M. neoaurum* (*2*)
*M. celatum* (*71*)	*M. nonchromogenicum* (*5*)
*M. chelonae* (*64*)	*M. novocastrense* (*2*)
*M. chimaera* (*4*)	*M. palustre* (*3*)
*M. chitae* (*2*)	*M. parascrofulaceum* (*3*)
*M. colombiense* (*3*)	*M. peregrinum* (*19*)
*M. conceptionense* (*1*)	*M. phocaicum* (*1*)
*M. cosmeticum* (*1*)	*M. scrofulaceum* (*19*)
*M. europaeum* (*1*)	*M. septicum* (*3*)
*M. flavescens* (*4*)	*M. shimoidei* (*4*)
*M. fortuitum* (*104*)	*M. simiae* (*18*)
*M. frederiksbergense* (*1*)	*M. smegmatis* (*4*)
*M. gastri* (*2*)	*M. szulgai* (*25*)
*M. genavense* (*11*)	*M. terrae* (*5*)
*M. gilvum* (*2*)	*M. triviale* (*1*)
*M. gordonae* (*527*)	*M. vaccae* (*2*)
*M. haemophilum* (*1*)	*M. ulcerans* (*2*)
*M. heckeshornense* (*4*)	*M. xenopi* (*140*)
*M. heidelbergense* (*3*)	

### Localization of NTM disease

The overall incidence of pulmonary- and non-pulmonary NTM cultures were 1.78/10^5^/year and 0.69/10^5^/year, respectively. However, adults and children differed. Adults aged 15 and above had an annual incidence rate from a pulmonary or non-pulmonary site of 2.14/10^5^ and 0.38/10^5^, respectively. Conversely, children had an annual incidence of non-pulmonary NTM cultures of 2.00/10^5^/year, whereas the incidence of NTM cultures from a pulmonary site was 0.20/10^5^/year. The relation between young age and extra-pulmonary NTM site was related to MAC infections; 95% of all clinical specimens containing MAC in children originated from lymph nodes. Conversely, among persons aged > 14 years, MAC was isolated from pulmonary sites for 75% of cases and from non-pulmonary sites for only 16% of cases (Table 1), whereas 9% were from multifocal sites. Localization of NTM disease also differed according to species. *M. marinum* was isolated from an extra-pulmonary site in 98% of the cases, while 1% was pulmonary and 1% multifocal. Conversely, *M. xenopi* was isolated from a pulmonary site in 95% of cases.

## Discussion

This is the largest nationwide study estimating the burden of NTM disease in a TB low incidence country to our knowledge. The study includes 524,119 mycobacterial cultures from all examined cases in Denmark through a quarter century. Based on this comprehensive and exhaustive dataset, we have evidence that contradicts the numerous reports of a general increase in NTM disease incidence^5, 8, 10, 14, 15^.

From 1991 through 2015, the annual incidence of culture-verified definite NTM disease and possible NTM disease remained steady. The overall incidence of positive cultures from pulmonary sites among adults was 2.14/10^5^/year; 1.10/10^5^/year for cases with definite- and possible NTM disease. The incidence of cultures from non-pulmonary sites among adults (all definite NTM disease cases) was 0.38/10^5^/year. These results are comparable to other studies in TB low incidence countries. In New Zealand, Freeman *et al*. found the pulmonary- and non-pulmonary NTM disease incidence to be 1.2/10^5^/year and 0.63/10^5^/year, respectively, while van Ingen reported an NTM disease incidence of 1.7/10^5^ in the Netherlands in 2008^16, 20^. In general, the incidence of NTM disease varies in different studies depending on study design, sample sites, diagnostic criteria, and geographical distribution, and different classifications of NTM disease versus NTM colonization is a challenge when comparing studies^9, 13, 21, 22^. Furthermore, compared to our study covering a quarter-century, previous studies investigate NTM disease trends based on shorter time intervals up to maximal 12 years, thus the reported incidence- and prevalence rates are more susceptible to random fluctuations^5, 8, 10, 14, 15^.

In children, our evidence corroborates that the clinical manifestations of MAC are very different from adults. The most common clinical presentation of NTM disease in children is cervical adenitis most often due to mycobacteria from the *M. avium* complex (MAC)^13^. This localized lymph node enlargement is a chronic infection different from the pulmonary disease observed among adults, and it is occurring in immunocompetent children aged 1–5 years without systemic symptoms^13^. In children < 15 years, 80% of all positive cultures were MAC infection, 95% of these from non-pulmonary sites. In contrast, among persons ≥ 15 years, MAC was identified in respiratory samples in 75% cases, 50% of these with definite NTM disease. The incidence of extra-pulmonary MAC disease in our study peaks in children aged 0–4 years, as seen in other studies^23, 24^. The high MAC incidence rates among children might reflect direct infection through the throat mucosa as children are more likely to have frequent direct contact with MAC sources in dirt, soil and water. Among adults, studies have shown that occupational exposure to soil is associated with an increased risk of MAC infections^25^. Also, a more immature cellular immunity in children could be part of the explanation; i.e. it has been suggested that T cell-dependant production of type 1 cytokines may be affected in children with lymphadenitis, and that the adenitis manifestation could be due to an impaired immunological response^26, 27^.

*M. marinum* originated from an extra-pulmonary site in the vast majority of cases (98%). Among these, the clinical specimen was a skin or wound biopsy in 90%, while the remaining sites were from a bone or joint biopsy. These findings confirm the presence of *M. marinum* as a pathogen prone to cause cutaneous infections^28^.

Elderly from age 65 had a relative risk of a positive NTM culture more than ten times that of adolescents aged 15–19 when we calculated age-standardized incidence rates. This association between old age and an increased incidence of NTM disease is well known and has been associated with comorbidities in the ageing population^29^. In addition, we found the median age of patients with a positive NTM culture to increase during the last 25 years. Initially, we hypothesized that increased longevity in the population was partly responsible for the expected increase in NTM incidence rates found in other studies, but our study does not support this hypothesis, as our main finding is that the incidence of NTM disease did not increase during the study period.

Additionally, the incidence of patients with NTM colonization, did not increase during the study period. It could be that the clinical approach to diagnosing NTM disease has not changed during the study period, as an increased attention to NTM disease would intuitively result in more cultures and thus probably more cases of both NTM colonization and NTM disease. The steady annual number of specimens cultured at the IRLM during the study period supports this hypothesis, except for the years 1998 and 1999, where high rates of NTM colonization were observed, the vast majority was due to *M. gordonae* (77% and 66% respectively), a known contaminant from tap water. This finding and subsequent decrease in clinical isolates containing *M. gordonae* is most likely due to an increased attention towards avoiding colonization replacing tap water with sterile water when performing diagnostic procedures.

Worldwide, the number of NTM disease cases surged in the eighties and nineties due to the HIV pandemic, when disseminated or extra-pulmonary Mycobacterial diseases often was seen as the AIDS-defining illnesses^30^. Later, after the introduction of highly active antiretroviral treatment (HAART) during 1996, there was a decline in AIDS-associated NTM disease^31^. To investigate HIVs potential influence on our results, we tried to exclude all NTM cases associated with AIDS by combining our data with the mandatory AIDS registry. Still, the incidence of definite NTM disease remained at a steady level during the study period (p = 0.854, see Table 5). Thus, the HIV pandemic did not influence our conclusions of no increase in NTM disease incidence.

**Table 5:** Number of HIV-positive patients where nontuberculous mycobacteria are the AIDS-defining illness.

	1991	1992	1993	1994	1995	1996	1997	1998	1999	2000	2001	2002	2003
AIDS and NTM disease	1	10	12	9	9	9	5	3	2	1	3	0	0
	**2004**	**2005**	**2006**	**2007**	**2008**	**2009**	**2010**	**2011**	**2012**	**2013**	**2014**	**2015**	
AIDS and NTM disease	2	3	1	5	0	0	0	1	0	1	0	1	

AIDS: Acquired Immune Deficiency Syndrome.

Our study holds some limitations. First, the assignment of positive cultures to one of the three disease categories was solely based on microbiological criteria from the adapted ATS/IDSA guidelines for diagnosing NTM disease^13^. We had no access to information on treatment and the physicians’ diagnostic criteria. However, our approach has been used and validated several times and proven useful for epidemiological studies^4, 12^. Second, we report annual incidence rates of culture verified NTM disease. Thus, only patients suspected of mycobacterial disease are investigated, and only those with a positive NTM culture included. Therefore, the calculated incidence rates might underestimate the true NTM burden in the Danish population. However, we find it unlikely that (many) patients are treated for NTM disease without any positive culture(s) whatsoever. Therefore, we believe our finding of no trend towards an increase in NTM disease during the study period is valid.

In summary, based on more than half a million clinical specimens analysed for mycobacteria during a quarter century in Denmark, we cannot confirm the numerous reports on increasing incidence of NTM disease. Our finding highlights the need for a systematic approach to diagnosing NTM disease, including adherence to existing diagnostic guidelines. We need a uniform reporting of NTM disease and more studies critically questioning the NTM disease epidemiology.
